# Hemoglobin E syndromes in Pakistani population

**DOI:** 10.1186/1471-2326-12-3

**Published:** 2012-03-25

**Authors:** Bushra Moiz, Mashhooda Rasool Hashmi, Amna Nasir, Anila Rashid, Tariq Moatter

**Affiliations:** 1 Department of Pathology and Microbiology, The Aga Khan University Hospital, Stadium road, Karachi 74800, Pakistan

**Keywords:** Hemoglobin E, Hemoglobin variants, Pakistan

## Abstract

**Background:**

Hemoglobin E is an important hemoglobin variant with a worldwide distribution. A number of hemoglobinopathies have been reported from Pakistan. However a comprehensive description of hemoglobin E syndromes for the country was never made. This study aimed to describe various hemoglobin E disorders based on hematological parameters and chromatography. The sub-aim was to characterize hemoglobin E at molecular level.

**Methods:**

This was a hospital based study conducted prospectively for a period of one year extending from January 1 to December 31, 2008. EDTA blood samples were analyzed for completed blood counts and hemoglobin variants through automated hematology analyzer and Bio-Rad beta thalassaemia short program respectively. Six samples were randomly selected to characterize HbE at molecular level through RFLP-PCR utilizing *Mnl*I restriction enzyme.

**Results:**

During the study period, 11403 chromatograms were analyzed and Hb E was detected in 41 (or 0.36%) samples. Different hemoglobin E syndromes identified were HbEA (n = 20 or 49%), HbE/β-thalassemia (n = 14 or 34%), HbEE (n = 6 or 15%) and HbE/HbS (n = 1 or 2%). Compound heterozygosity for HbE and beta thalassaemia was found to be the most severely affected phenotype. RFLP-PCR utilizing *Mnl*I successfully characterized HbE at molecular level in six randomly selected samples.

**Conclusions:**

Various HbE phenotypes are prevalent in Pakistan with HbEA and HbE/β thalassaemia representing the most common syndromes. Chromatography cannot only successfully identify hemoglobin E but also assist in further characterization into its phenotype including compound heterozygosity. Definitive diagnosis of HbE can easily be achieved through RFLP-PCR.

## Background

Hemoglobin E is an important and common β-globin chain variant resulting from substitution of glutamine by lysine at codon 26 of β-globin gene (∞2β2 ^26Glu→Lys^). Since its first description by Chernoff and his colleagues in 1954 [1], HbE was increasingly reported from several parts of the world. Hb E confers a survival advantage against *Plasmodium falciparum *and could be the logical explanation for its high global prevalence [2]. It is the most prevalent abnormal hemoglobin in South East Asia with its frequency approaching 60% in Northeast regions of Thailand [3,4], Laos and Cambodia [5]. Significant numbers were reported from other Asian countries such as Sri Lanka [6], North Eastern India [7], Bangladesh [8], Nepal [6], Vietnam [9] and Malaysia [10]. Additionally, population transmigration led to its emergence in United States [11] and Canada [5].

Hb E occurs both in homozygous (EE) and heterozygous (EA or E trait) states and may co-inherits with alpha [12] and beta thalassaemia [13], HbS, HbC and other hemoglobin variants [6]. Hb EE and E trait are mild disorders and are associated with either mild or no anemia. In contrast, HbE/beta thalassaemia displayed a remarkable variability in its clinical severity varying from mildly asymptomatic state to a severe transfusion dependent anemia [14]. HbE/HbS results in a sickle cell disorder similar to sickle/beta + thalassaemia [14].

It is important to diagnose Hb E disorders correctly as the clinical course is variable for various phenotypes and would modify the management. A cost effective and simple combination of hemoglobin instability and osmotic fragility tests was devised for detection of Hb E in low socio-economic countries [15] Other methods include hemoglobin electrophoresis where Hb E runs with the same speed as Hb A_2 _on alkaline pH requiring subsequent identification on acidic pH. On high performance liquid chromatography (HPLC), Hb E has the same retention time as HbA_2 _[16] but is identified because of its quantity which is substantial in comparison to HbA_2_. Ultimate diagnosis of this variant rests on molecular analysis. RFLP-PCR [17], DNA sequencing and allelic discrimination analysis [18]are successfully used for molecular characterization of hemoglobin E.

Situated in South Asia, Pakistan is a home to 180 million people. Various hemoglobinopathies have been reported from this country [19] including alpha [20] and beta thalassaemia [21,22], sickle cell disorders [23], Hb D [24,25], Hb Q [26]and other rare variants [27]. Sporadic cases of HbE have been reported from Pakistanis living in United Kingdom [6,28] but the information regarding HbE for the country remains fragmentary hitherto. Since Pakistan showed a high incidence of beta thalassaemia major [22,29], high estimates for HbE/β- thalassaemia syndromes were expected. Further to this credence, there was a need to characterize HbE in the large and diverse population of the country in order to ensure optimal patient management. The present hospital based study was focused on the identification of Hb E through HPLC and on stratification of its various phenotypes based on hematological parameters. No attempt was made to analyze other hemoglobinopathies as a number of previous studies had already addressed this issue. The study also aimed for molecular characterization of HbE.

## Methods

### Sample collection

We prospectively analyzed blood samples during one year from January 1 to December31, 2008 in the clinical laboratory of The Aga Khan University, Pakistan. The patients were referred by various physicians from within and outside the hospital for the work up of anemia, antenatal and pre-marital screening. The laboratory served as a referral laboratory for the country and samples were obtained from 94 blood collection centers within the city and 82 similar centers from the entire country. Clinical details were assessed based on information provided by the requesting physicians.

### Complete blood counts and chromatographic analysis of hemoglobin variants

Briefly, we collected five milliliters of blood from each patient in ethylene di-amine tetra acetate micro cuvettes (BD, Becton Dickinson and Company, New Jersey, USA). The samples were received cooled at 4°C by fast transportation in our lab and were analyzed within 24 h. Hemogram was performed on each sample by Coulter Gen-S (Coulter Electronics, Fullerton, CA, USA), and peripheral film stained with Leishman's stain was examined by experienced technologists and hematologists.

We used the Bio-Rad Variant Classic β Thalassaemia Short program (Bio-Rad Laboratories Inc. Hercules, CA, and USA) for the hemoglobin quantification. This automated system utilizes the principle of cation exchange HPLC with detection at double wavelength (415 and 690 nm). The instrument is user friendly and analyzed each sample for a period of 5-6 min., with various positively charged hemoglobin molecules eluting at different times depending on their affinity for anion-coated resin columns. We use a retention time (RT) of 3.65 to 3.7 min to identify Hb E + Hb A_2 _as the two co-elute in the same A_2_window [16].

### Molecular analysis

**Figure 1:**
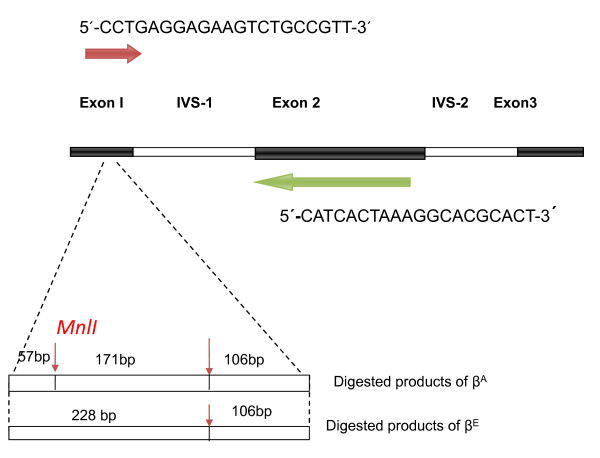
**Schematic representation of β- globin gene and sequence amplified by forward primer (red) and reverse primer (green) and the cut DNA in β^A ^and β^E ^globin gene subsequent to digestion with restriction enzyme *Mnl*I (red thin arrow)**.

In mutational analysis, we prospectively selected 6 samples which were screened as Hb E trait (n = 2) and Hb E/beta thalassaemia (n = 4) on HPLC. Total genomic DNA was isolated from peripheral white cells according to manufacturer's instructions using Wizard^® ^Genomic DNA Purification kit (Promega USA Cat No: A1125). RFLP-PCR based approach was used for detecting β26 G → A mutation utilizing enzyme *Mnl*I [30]. Hb E mutation destroys a cleavage site for *Mnl*I which cleaves DNA at the sequence 3'-GAGG-5' [31]. The schematic representation of beta gene with primers and sequence amplified along with cut base pair sizes are detailed in Figure 1. PCR amplification was performed using 1 μg of genomic DNA in 25 μl reaction optimized by using the following concentrations: 5 × buffer 5 μl, MgCl2 1.5 μl, DNTP 1 μl, each primer 0.5 μl, Taq polymerase 0.5U reconstituted with 14 μl of water. Eppendorf ^®^Master Cycler gradient (Germany) using annealing temperatures calculated according to Tm of primer pairs run at 34 cycles with a further 7 min extension at 72°C. Amplified products were digested with 5 units of *Mnl*I and separated through electrophoresis on 1.5% agarose gel. These were visualized under UV light after ethidium bromide staining.

### Statistical analysis

The data was entered into SPSS version 16(SPSS Inc., Chicago, IL, USA) to compute descriptive data. Various hemoglobin E phenotypes were identified with respect to hemoglobin, red cell indices and hemoglobin variants (HbE + HbA_2_, Hb F, HbA or any other variant) as described in literature [6,14,18].

### Ethical issues

The study was approved by institutional ethical review committee of The Aga Khan University (ERC approval #1600-P80-ERC-2010). The retrieved computerized data from laboratory was re-coded to maintain anonymity. The samples were collected from patients after their informed consent.

## Results

We reviewed 11403 chromatograms in the year 2008 identifying 1944 (17%) samples with hemoglobin variants. The hemoglobinopathies detected were as follows: β -thalassaemia trait, β -thalassaemia major, Hb S disorders, Hb D and Hb Q in 1174 (60%), 274(14%),246 (13%),193 (10%) and 7(0.4%) samples respectively. One case of Hb C and Hb H each were identified. Another 48 cases (2% of abnormal variants or 0.4% of total analyzed samples) were observed having the retention time of 3.65 to 3.7 min and were preliminary labeled as Hemoglobin E. There were seven samples with 38-48% of abnormal hemoglobin where the possibility of Hb D Iran could not be excluded. These were excluded from final analysis leaving a total of 41 cases for evaluation of Hb E.

### Subjects characteristics

The mean age (± SD) was 13.6 years (± 11.3) ranging from 4 months to 38 years. There were 23 males and 18 females. Clinical details were available for some of the patients and hence were discussed where obtainable.

### Hematological analysis

#### 1. Hb E Trait

This constituted the largest diagnosed group comprising of 48.8% (n = 20). There were 10 males and an equal number of females with a median age of 14.0 ± 12.8 years. Thirteen children and seven adults were diagnosed. Hemoglobin E ranged from 16.4 to 30.6% (mean ± SD; 25.9 ± 4.3). Six patients showed high HbF ranging from 4.1 to 8.6%. Only six (30%) subjects (four females and two males) had hemoglobin within reference range. Remaining 14 patients had lower mean hemoglobin, MCV and MCH [8.6 ± 2.4 g/dl, 65.5 ± 9.1 fl and 21.2 ± 3.4 pg respectively] as well as hypochromic microcytic red cells with anisocytosis. Three patients had severe anemia with hemoglobin less than 7.0 g/dl.

#### 2. β-thalassaemia/HbE

Fourteen (34%) cases (10 females and 4 males with median age of 9.3 ± 7.3 years) were diagnosed as compound heterozygotes for HbE and β- thalassaemia. None of the patient in this group showed hemoglobin above 8 g/dl. The mean MCV and MCH were 68.1 ± 9.3 fl and 21.0 ± 3.3 pg respectively. Hb F was high in all patients ranging from 16.3 to 64.7% while HbE was variable from 11.6 to 65.3%. Peripheral film review showed hypochromic microcytic red cells in all cases with nucleated red cells in twelve cases. No attempt was made to differentiate HbE/β° and HbE/β + as samples were not analysed for β-thalassemia mutations.

#### 3. HbEE Homozygosity

Hemoglobin EE disorder was observed in six (14.6%) patients (four males and two females with median age of 24 ± 8 years). The criteria for diagnosis was a high HbE (> 78%) and a low HbF (< 3%). The females (n = 4) had hemoglobin in the range of 9.6 to 10.6 g/dl. Two males aged four and 25 years had hemoglobin of 7.6 g/dl each. All subjects had hypochromic and microcytic red cell indices. Only one patient had high HbF (8.5%) then the set criteria. He was a 25 year old male with hemoglobin of 7.6 g/dl. The possibility of co-existing α-thalassaemia cannot be excluded in this patient.

#### 4. HbE/Hb S

Compound heterozygosity for HbE and HbS was seen in one case. This patient presented at the age of two years with anemia and splenomegaly and had received only one transfusion in the past two years. Hematological parameters are given in Table 1.

**Table 1:** Hematological features of various hemoglobin E phenotypes (n = 41)

Phenotype	n	Hb (g/dl)	MCV (fl)	MCH (pg)	HbA (%)	HbE + A2 (%)	Hb (F %)	Others (%)
EE	6	8.8 ± 1.6	56.9 ± 4.1	17.4 ± 1.8	0	81.7 ± 1.9	2.4 ± 3.3	-
EA	20	9.7 ± 2.6	67.3 ± 8.1	21.9 ± 3.1	72.3 ± 4.3	25.9 ± 4.3	2.1 ± 3.4	-
E/β-thalassaemia	14	5.0 ± 1.8	68.1 ± 9.3	21.0 ± 3.3	19.9 ± 16.0	43.7 ± 16.3	36.3 ± 13.8	-
E/S	1	7.6	67.6	20.4	0	11.8	19.3	HbS = 68.9
**All cases**	**41**	**7.9 ± 3.0**	**66.0 ± 8.7**	**20.9 ± 3.3**	**42.1 ± 32.1**	**39.8 ± 21.9**	**14.2 ± 18.3**	

### Molecular analysis

**Figure 2:**
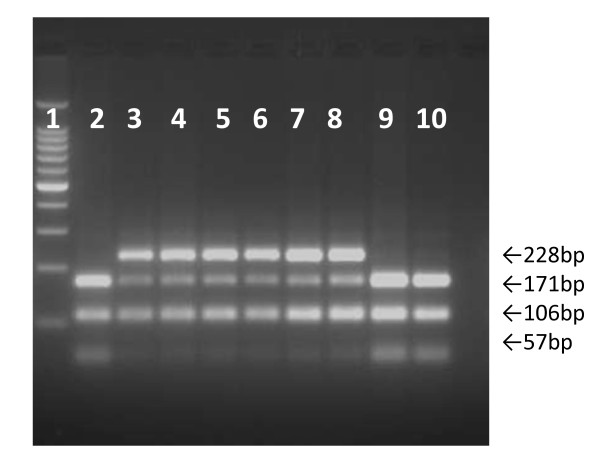
**Cut DNA subsequent to digestion with *Mnl*I**. M = Molecular marker of 100 bp, lanes 1, 8, 9 = normal subjects; lane 2 and 3 = subjects heterozygous for HbE (β^A^/β^E^); lanes 4-7 = subjects compound heterozygous for HbE and beta thalassaemia; lane 10 = blank.

Results of DNA analysis of six samples before and after *Mnl*I digestion are shown in Figure 2. The amplification of gene produces a 334 bp product in wild and mutant types (figure not shown). Subsequent to digestion three fragments of 57, 106 and 171 bp were observed in wild type and two fragments 106 and 228 bp products were seen in mutant type. Two heterozygous subjects (β^A^/β^E^) and four with compound heterozygosity for β thalassaemia/HbE showed four fragments of 57, 106, 171 and 228 bp.

## Discussion

Hb E was detected in 0.4% of our samples. Our clinical lab receives samples from the entire country hence the population was well represented in the study. Since this was a hospital based study, the observed frequency might be an overestimation of the true incidence in general population. However, our intention was to identify and report HbE in Pakistani population and this study served our purpose. Heterozygous AE represented the commonest phenotype followed by β- thalassaemia/Hb in Pakistan. Various HbE phenotypes were differentiated through cation exchange chromatographic analysis and successfully characterized at molecular level. For diagnosis of HbE, a retention time of 3.65-3.7 min was used. Besides HbE, several hemoglobin variants [HbA_2_, Hb Lepore, Hb D-Iran, HbG-Honolulu, Hb Korle-Bu] elute in the A_2 _window(RT: 3.30-3.90) [16]. These can be successfully differentiated on the basis of variance in their quantities and RT [16]. Hence authors believe that HbE was correctly identified as supported by the molecular studies.

Clinically HbE is a mild type of disorder both in homozygous and heterozygous states therefore Hb E individuals are minimally anemic and asymptomatic [6]. However, co-inheritance of hemoglobin E and beta thalassaemia trait results in β- thalassaemia/HbE disease having variable phenotypes ranging from transfusion dependence to a complete absence of symptoms [28]. This inconsistent expression is attributed to HbE instability during fever and oxidative stress leading to fall in hemoglobin concentration [32]. It is the commonest form of severe thalassaemia in the world and is most prevalent in South Asia. We observed that our subjects with β- thalassaemia/HbE were mainly young children (aged four months to 13 years) who presented with severe hypochromic and microcytic anemia having target cells, basophilic stippling, fragmented red cells and nucleated red cells. The young age at presentation reflects the severity of anemia and hence early consultation. This was the most severely affected phenotype observed and the patients in this group demonstrated very low levels of hemoglobin. Since, β-thalassaemia is widely prevalent in Pakistan with a carrier rate of 5% [22]; this combination of HbE and β-thalassaemia was not unexpected. We could not differentiate HbE/β° from HbE/β + as samples were not analyzed for β-thalassemia mutations. However, Rees DC studied 45 patients with β- thalassaemia/HbE and showed that HbE/HbF ratio was significantly higher (3.1) in patients with regular transfusions in contrast to 1.8 in un-transfused patients. This reflected the relative reduction in γ-globin chain synthesis subsequent to blood transfusions. HbE/HbF ratio in 12 of the patients in our study ranged from 0.27 to 2.0 (mean ± 1SD; 1.4 ± 0.98) showing infrequent blood transfusions. Two patients had higher ratios 2.65 and 4.0 but both of them were on regular blood transfusions.

Subjects with hemoglobin E trait were mostly young adults and the reasons for their testing were extended family screening or investigation of hypochromic microcytic red cells on routine CBC. Females were evaluated as a work up of their antenatal screening. Contrary to usual finding of mild or no anemia in hemoglobin AE, 14 subjects (70%) presented with hypochromic and microcytic anemia including three patients with severe anemia (< 7 g/dl). Two possible explanations were either the presence of iron deficiency anemia or concomitant occurrence of α- thalassaemia. Iron deficiency is rampant in Pakistan [33-35] while α- thalassaemia has a reported prevalence rate of over 2% in the country [20]. In 1987, Katsanis E et al. studied 33 children with HbE trait and observed co-existing anemia in 62% of them having substantially lower hemoglobin levels and HbE% than expected [5]. Unfortunately, iron studies and α- thalassaemia tests were not available for our subjects. Usually the carrier state demonstrates 20-30% of hemoglobin E but we observed a lower range of 16-19% in three cases. Authors believed that concomitant iron deficiency or alpha thalassemia resulted in lowering of Hb E [6].

Six cases with homozygous E were observed in the study. Our criteria of diagnosis were HbE > 75% and < 3% hemoglobin F. It has been argued that HbEE and HbE/beta thalassaemia are overlapping syndromes and cannot be phenotyped correctly [18,36]. Correct diagnosis is the key to the optimum management. However, our experience showed that HPLC can help in differentiating the two as HbF and HbE levels were significantly different in both phenotypes. Also, it is known that regular transfusions in HbE/beta thalassaemia may decrease HbF levels but not HbE [28] which could further assist in correct diagnosis.

We observed a single case of compound heterozygosity for hemoglobin E/S. Hemoglobin S had been reported from this region [23] and the occurrence of this combination was no surprise. However, HbE and F levels did not correspond to the previously published reports in the similar condition. We hypothesized that co-existing iron deficiency reduced the quantity of HbE and high HbF in sickle cell disorders is not atypical for this geographical region [23].

### Strengths and limitations

This was the first report describing the various Hb E phenotypes in Pakistan. The study was limited by patchy clinical details, lack of iron studies and molecular confirmation of α and β- thalassaemia. Being a single hospital based study; our data did not reflect the true prevalence of Hb E in Pakistani population.

## Conclusions

Various HbE phenotypes are prevalent in Pakistan with HbEA and HbE/β thalassaemia representing the most common syndromes. Chromatography cannot only successfully identify hemoglobin E but also assist in further characterization into its phenotype including compound heterozygosity. Definitive diagnosis of HbE can easily be achieved through RFLP-PCR.

## Competing interests

The authors declare that they have no competing interests.

## Authors' contributions

BM conceived of the study, participated in its design and coordination and wrote manuscript. MRH participated in study design, performed chromatography and analysis of the data. AN performed the mutational analysis and wrote part of the manuscript. AR collected the samples, wrote and conceived grant for molecular analysis and helped drafting of manuscript. TM participated in study design, conception and drafting of manuscript. All authors read and approved the final manuscript.

## Pre-publication history

The pre-publication history for this paper can be accessed here:

http://www.biomedcentral.com/1471-2326/12/3/prepub
